# Laminar Thickness Alterations in the Fronto-Parietal Cortical Mantle of Patients with Attention-Deficit/Hyperactivity Disorder

**DOI:** 10.1371/journal.pone.0048286

**Published:** 2012-12-11

**Authors:** Elseline Hoekzema, Susana Carmona, J. Antoni Ramos-Quiroga, Vanesa Richarte Fernández, Marisol Picado, Rosa Bosch, Juan Carlos Soliva, Mariana Rovira, Yolanda Vives, Antonio Bulbena, Adolf Tobeña, Miguel Casas, Oscar Vilarroya

**Affiliations:** 1 Unitat de Recerca en Neurociència Cognitiva, Departament de Psiquiatria i Medicina Legal, Universitat Autònoma de Barcelona, Bellaterra (Barcelona), Spain; 2 Grup de Recerca en Neuroimatge, Fundació IMIM, Barcelona, Spain; 3 Harvard Social Cognition and Affective Neuroscience Lab, Harvard University, Cambridge, Massachusetts, United States of America; 4 Department of Psychiatry, Hospital Universitari Vall d'Hebron, CIBERSAM, Barcelona, Spain; 5 Departament de Psiquiatria i Medicina Legal, Universitat Autònoma de Barcelona, Bellaterra (Barcelona), Spain; 6 CRC Corporació Sanitaria, Barcelona, Spain; 7 Port d'Informació Científica (PIC), Universitat Autònoma de Barcelona, Bellaterra (Barcelona), Spain; 8 Institut de Física d'Altes Energies, IFAE, Universitat Autònoma de Barcelona, Bellaterra (Barcelona), Spain; 9 Institut de Neuropsiquiatria i Adiccions, Hospital del Mar, Barcelona, Spain; Centre Hospitalier Universitaire Vaudois Lausanne - CHUV, UNIL, Switzerland

## Abstract

Although Attention-Deficit/Hyperactivity Disorder (ADHD) was initially regarded as a disorder exclusive to childhood, nowadays its prevalence in adulthood is well established. The development of novel techniques for quantifying the thickness of the cerebral mantle allows the further exploration of the neuroanatomical profiles underlying the child and adult form of the disorder. To examine the cortical mantle in children and adults with ADHD, we applied a vertex-wise analysis of cortical thickness to anatomical brain MRI scans acquired from children with (n = 43) and without ADHD (n = 41), as well as a group of adult neurotypical individuals (n = 31), adult patients with a history of stimulant treatment (n = 31) and medication-naïve adults with ADHD (n = 24). We observed several clusters of reduced laminar cortical thickness in ADHD patients in comparison to neurotypical individuals. These differences were primarily located in the dorsal attention network, including the bilateral inferior and superior parietal cortex and a section of the frontal cortex (centered on the superior frontal and precentral gyrus bilaterally). Further laminar thickness deficits were observed in the bilateral orbitofrontal cortex and medial occipital cortex. The deficits in the cortical surface were especially pronounced in the child sample, while adult patients showed a more typical laminar thickness across the cerebral mantle. These findings show that the neuroanatomical profile of ADHD, especially the childhood form of the disorder, involves robust alterations in the cortical mantle, which are most prominent in brain regions subserving attentional processing.

## Introduction

Attention-Deficit/Hyperactivity Disorder (ADHD) is one of the most common psychiatric disorders of childhood, affecting approximately 8–12% of school-age children worldwide [1]. This childhood-onset disease is characterized primarily by symptoms of inattention, hyperactivity and impulsivity that interfere with normal functioning in various settings [2]. Contrary to the initial conception about ADHD as a disorder exclusive to childhood, nowadays its persistence into adolescence and adulthood is well established. Studies in the adult population indicate that around 35% of the pediatric patients still fulfill ADHD diagnostic criteria in adult life [3].

Initial studies investigating the neural bases of the disorder depended on the manual (or semi-manual) delineation of the whole encephalon or of a priori defined regions of interest (ROIs) by trained neuroanatomists. The first study relying on this methodology in the early twentieth century detected a reduction of total brain volume in children with ADHD [4], a finding that has since then been extensively replicated. Furthermore, manual ROI segmentation studies have uncovered localized grey matter volume decreases in the frontal lobe, basal ganglia (including the caudate, putamen and globus pallidus) and cerebellum (especially the posterior inferior lobules and cerebellar vermis). These findings strengthened the hypothesis that alterations in fronto-striato-cerebellar circuits sustaining executive functions play an important role in the pathophysiology of ADHD. Although less discussed, abnormalities in other brain regions such as the parieto-occipital cortex were also repeatedly identified using classical segmentation approaches [5].

The manual segmentation technique, which is considered a gold standard in terms of its anatomical validity, is a very labor-intensive procedure that has to contend with problems of inter - and intra-rater reliability. Furthermore, findings derived from this methodology are unavoidably biased by the a priori definition of brain regions expected to show volumetric alterations. Traditional methods were soon complemented by automatic and highly replicable procedures, such as voxel-based morphometry (VBM), which are less time-consuming and allow the simultaneous assessment of whole-brain cortical and subcortical structures. Studies applying VBM not only confirmed cerebellar and basal ganglia alterations but also detected deviations in other sections of the brain, especially in the cortical mantle. VBM studies in children with ADHD revealed smaller grey matter volumes across several cortical regions. Reduced volume in the frontal and parietal cortex – especially in the superior/middle frontal gyrus, orbitofrontal cortex, premotor cortex and inferior parietal cortex— are among the most replicated [6]–[11]. However, volumetric decreases have also been observed in the temporal cortex (particularly in the superior temporal sulcus, middle temporal cortex and temporal pole) as well as in various regions within the occipital cortex [6], [9], [12], [13]. These findings highlighted the presence of widespread alterations in the cortical mantle in patients with ADHD, mostly localized to regions known to subserve executive functions and attentional processing.

The evidence of substantial cortical alterations in ADHD underscored the necessity to complement VBM techniques with other sophisticated methods, such as analyses of cortical thickness, which are devoted to specifically map differences in laminar thickness between diagnostic groups. Although VBM is a powerful tool to examine volumetric tissue properties, the amount of grey matter density or volume as estimated by VBM can be confounded by how convoluted the brain is in a given region. Cortical thickness analyses are commonly based on the estimation of the distance between the grey-white matter border and the pial surface, thus they use the local topography of the grey matter to generate a specific and quantifiable metric of a cortical anatomical property. Consequently, measuring the thickness of the cortex provides a close approximation of the underlying anatomical reality. Compared to volumetric measures, cortical thickness procedures are less susceptible to positional variance [14], [15]. The main disadvantage of cortical thickness procedures compared to VBM methods is that the former cannot provide information about subcortical structures since the analysis is restricted to the cortical mantle.

The few previous studies that have assessed cortical thickness in children and adolescents with ADHD obtained results that largely echoed those observed using automatic volumetric approaches [16]–[25]. Decreased cortical thickness has been reported in regions within the frontal cortex (particularly in the right inferior frontal gyrus, precentral cortex and orbital areas), the lateral association parietal cortex (including both the superior and inferior parietal cortex), the temporal pole and the ventral parts of the temporo-occipital junction mainly involving the lingual and fusiform gyri. Interestingly, some of the reported decreases in the cortical mantle seem to result from delays in cortical development [18], [20], [22]. For instance, Shaw et al. demonstrated that normal developmental trajectories for reaching peak cortical thickness are delayed in ADHD across most of the prefrontal and lateral temporal cortex, and clusters of thinner cortex in the right inferior parietal cortex observed in ADHD patients during childhood tend to normalize by the late teenage years in those participants with a better clinical outcome [18], [20], [22]. It should be noted, however, that these longitudinal studies are based on medicated samples and did not include subjects older than 18 years.

Although several studies have applied cortical thickness analyses to children with ADHD [16]–[25], the cortical mantle in adult patients remains relatively unexplored. The first study investigating cortical thickness in adult ADHD patients, performed in 2007 by Makris et al., observed a thinner cortex in nearly all a priori regions that subserve attention and executive functions, including the bilateral dorsolateral prefrontal, orbitofrontal, anterior/posterior cingulate cortex, and the right lateral inferior parietal regions, angular gyrus and supramarginal gyrus [26]. More recently, Almeida et al. compared the cortical thickness of the frontal lobe using a cross-sectional study of children, adolescents and adults and found that individuals with ADHD, regardless of age, had a significantly thinner right superior frontal cortex in comparison to controls [16]. They also performed a whole-brain cortical thickness analysis on this sample, and found smaller thickness measures primarily in fronto-parietal regions, while increased laminar thickness was measured in the occipital lobe [27]. In addition, a prospective follow-up study of patients with ADHD showed that adults who were diagnosed with ADHD during childhood, regardless of the current diagnosis, had thinner cortex in frontal regions as well as in the temporal poles and the inferior parietal lobe in comparison to control subjects [13]. Finally, Duerden et al performed a ROI-based analysis focusing on sensorimotor regions in adolescents and adults with ADHD and they observed increased cortical thickness in these regions [28]. Altogether, these observations suggest that certain cortical thickness deficits persist into adulthood, at least in individuals that remain symptomatic in adult life.

To expand upon earlier findings and further explore cortical alterations associated with the adult and child forms of ADHD, we carried out a cross-sectional study of cortical thickness comparing both adults and children with ADHD to a group of neurotypically developed controls. We used a vertex-wise analysis of cortical thickness that allows for an exploratory data analysis of the laminar thickness across the cortical mantle without placing a priori constraints on the search space for anatomical alterations.

## Methods

### Ethics Statement

The study was approved by the Hospital Universitari Vall d'Hebron ethics committee, and written informed consent was obtained from the subjects and from the parents or guardians of the child participants prior to their participation in the study.

### Subjects

For this study, 188 scans were collected from previous studies performed in our research group [29]–[35]. These subjects had been recruited from the Servei de Psiquiatria at Vall d'Hebron Hospital Universitari. Control subjects had been selected either from the traumatology department (patients with minor physical trauma), or by local advertisements. All subjects had been carefully evaluated in order to exclude comorbidity with other psychiatric or personality disorders. Persons that met diagnostic criteria for substance use disorder of drugs (including cocaine, heroin or synthetic drugs) or alcohol at any point in their life were excluded from the study. All patients fulfilled diagnostic criteria for ADHD (in both adults and children onset of symptomatology commenced before the age of 7 years). ADHD diagnosis was based on the Diagnostic and Statistical Manual of Mental Diseases, Fourth Edition, Text Revision (DSM-IV TR) [2]. See Table 1 for demographic data of the participants. Further clinical and demographic data of the samples and the acquisition parameters are described in other papers [29]–[35].

**Table 1:** Demographic data of the participants.

	CHILDREN		ADULTS	
	ADHD	Controls	ADHD	Controls
**Group size**	43	41	55	31
**Age (mean±s.d.)**	11.60±2.90	11.22±2.96	31.98±10.69	30.29±8.23
**Gender**	35 M, 8 F	28 M, 13 F	55 M, 0 F	31 M, 0 F
**Medication exposure**	38 MD, 5 MN	-	31 MD, 24 MN	-

These data were collected from different studies performed at our research group over the last 7 years. Several other clinical and demographic measures such as indices of symptom severity and IQ have not been included as they were collected using divergent instruments across the different studies. Further clinical and demographic data of the samples and the acquisition parameters are described in other papers [29]–[35]. M = Male; F = Female; MD = Medicated; MN = Medication-Naïve.

After initial processing, eighteen subjects had to be removed from further analyses due to insufficient quality of the white and pial surfaces resulting from the tessellation. Our final sample consisted of 86 adults (55 patients and 31 control subjects) and 84 children (43 patients and 41 control subjects). In the adult group, 24 patients had never received any pharmacological treatment for their condition, while only 5 of the child patients were medication-naïve.

### Cortical thickness analysis

The analyses were performed in Freesurfer (http://surfer.nmr.mgh.harvard.edu/) implemented in the PICNIC platform (http://neuroweb.pic.es/), using a standard cortical thickness approach. Cortical thickness analyses have previously been validated using histological measures in human brains [36], and a good test-retest reliability has been demonstrated across different field strengths, scanner upgrades and manufacturers [37].

The data were first normalized to a standard anatomical template [38] and corrected for bias-field inhomogeneities. Then the brains were skull-stripped using a watershed algorithm and subsequently segmented into white matter and non-white matter partitions. The initial tessellation was formed by reconstructing the grey matter/white matter boundary (white surface) and the outer cortical surface (pial surface). The resulting images of each individual were visually inspected and manually corrected and re-inspected if necessary. The thickness across the cortical mantle was extracted by computing the distance between the white and pial surfaces. To visualize the results, we projected the generated clusters onto pial and inflated surfaces and smoothed the data on the surface using iterative nearest-neighbor smoothing (10 mm FWHM).

We first compared the patient and control subjects in the adult and child sample (including both the medication-naïve patients and patients with a history of stimulant drug treatment in the patient groups). To evaluate whether the child and adult form of the disorder are associated with a distinct neuroanatomical profile, we also performed an ANOVA and applied the interaction contrast ‘Age Group×Diagnosis’. To explore the effects of stimulant medication on cortical thickness, we performed some additional analyses separately for the medicated and never-medicated patients. The two sample t-tests comparing the patients with the control participants were repeated for both the adult and the child sample after excluding the medication-naïve patients. Furthermore, for the adult subjects, we also compared cortical thickness in the medication-naïve group of adult patients with ADHD with the medicated patient group. The interaction contrast (‘Age Group×Diagnosis’) was also repeated without the medication-naïve patients in both groups.

To evaluate whether there are any effects of gender in the ADHD and control group besides the normal differences between male and female brains, we performed an ANOVA assessing the interaction between diagnosis and gender on the child sample (the adult sample did not include any female participants). Moreover, to ensure that subtle differences in gender distribution between the adult and child groups did not underlie the observed differences in cortical thickness between the samples, we also repeated the interaction contrast excluding the 21 female participants from the child control and ADHD groups.

To correct for multiple comparisons, we applied the monte carlo test simulation bootstrap (MCTSB) at a threshold of p<0.05. For completeness, when no differences were observed at this threshold, we reported the results at a threshold of p<0.001 uncorrected for multiple comparisons. In these cases, it is clearly indicated that the threshold was lowered for these comparisons and that no results were obtained at a corrected threshold.

## Results

**Figure 1:**
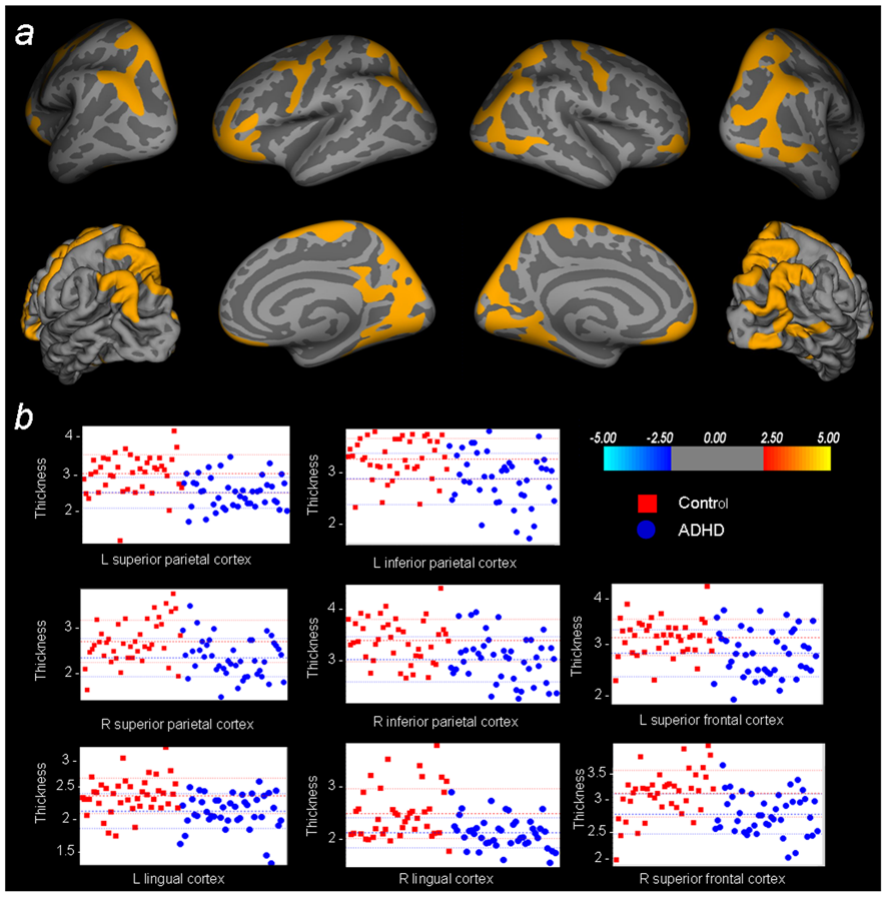
Whole-brain cortical thickness comparisons child sample. A) Statistical maps of the contrast ‘Control child>ADHD child’ (colors encode the −log10(p-value)), overlaid on inflated and pial brain surfaces. The results are thresholded at p<0.05, MCTSB corrected for multiple comparisons. B) Plots depicting the thickness values of the participants for the peak vertex of the primary clusters resulting from this comparison.

To evaluate the neuroanatomical profile associated with the child form of the disorder, we examined the thickness of the cortical mantle in the group of ADHD children in comparison to the child control sample. When comparing these groups, we observed substantial deficits in laminar thickness across the cerebral mantle, primarily localized in sections of the fronto-parietal cortex (see Table 2). More specifically, clusters of reduced laminar cortical thickness were observed in the bilateral inferior and superior parietal cortex, as well as in a section of the frontal cortex extending from the superior frontal gyrus to the precentral gyrus. Furthermore, patients had a thinner cortical mantle in the orbitofrontal cortex and a bilateral section of the medial occipital cortex, primarily covering the lingual gyri. Figure 1B illustrates individual thickness values within the observed clusters of thinner cortical surface.

**Table 2:** Cortical thickness comparisons child sample.

Contrast	Cortical region	Hemisphere	Talairach			Size	p_max_
			X	Y	Z	(mm^2^)	
**All ADHD vs Control**							
Control>ADHD	Inferior Parietal*	L	−47.4	−58.1	41.0	7817.34	1.16E-07
	Superior Parietal*						
	Cuneus*						
	Inferior Parietal*	R	36.1	−74.6	33.1	12932.79	4.80E-10
	Superior Parietal*						
	Cuneus						
	Lateral Occipital						
	Middle Temporal						
	Lingual*						
	Fusiform						
	Lingual*	L	−6.3	−80.8	0.2	3190.14	1.98E-05
	Fusiform						
	Precentral*	L	−39.0	−5.4	54.0	2126.83	1.27E-06
	Superior Frontal*						
	Caudal Middle Frontal						
	Precentral*	R	46.5	−5.2	44.4	2797.57	2.70E-06
	Superior Frontal*						
	Orbitofrontal *	L	−35.1	38.1	−10.4	5547.78	2.79E-06
	Rostral Middle Frontal*						
	Inferior Frontal						
	Orbitofrontal	R	19.6	60.4	−3.3	2421.24	2.14E-05
	Rostral Middle Frontal						
Control<ADHD	-						
**Medicated ADHD vs Control**							
Control>ADHD	Inferior Parietal*	L	−47.4	−58.1	41.0	7577.90	2.12E-07
	Superior Parietal*						
	Precuneus						
	Cuneus						
	Superior Frontal						
	Inferior Parietal*	R	36.1	−74.6	33.1	12534.43	3.99E-10
	Superior Parietal*						
	Superior Frontal*						
	Cuneus						
	Lateral Occipital						
	Inferior Temporal						
	Lingual*						
	Fusiform						
	Lingual*	L	−7.6	−81.6	−0.3	2654.72	7.33E-05
	Fusiform						
	Precentral*	L	−38.4	−5.5	53.6	2767.74	2.17E-06
	Postcentral						
	Orbitofrontal*	L	−35.3	37.5	−10.2	2677.03	5.96E-06
	Rostral Middle Frontal						
	Superior Frontal						
	Inferior Frontal						
	Orbitofrontal	R	9.1	29.9	−19.2	1451.53	1.91E-04
	Rostral Middle Frontal						
Control<ADHD	-						

Full results of the ‘control>ADHD’ comparison in the child groups including all ADHD subjects. The second section of the table provides the results of the same comparisons including only those patients with a history of pharmacological treatment with psychostimulant medication. Results are thresholded at p<0.05, MCTSB corrected for multiple comparisons. No clusters of thicker cortical mantle were observed in the ADHD sample compared to the control children (contrast ‘control<ADHD’).

* Clusters in these regions also survive a threshold of p<0.001, MCTSB corrected for multiple comparisons.

Then, we compared the adult ADHD patients – combining the medication-naïve sample and the patients with a history of pharmacological treatment- to the group of neurotypically developed adults. At the threshold of p<0.05 MCTSB corrected for multiple comparisons, however, we found no differences in cortical laminar thickness between the groups. Only when lowering the applied threshold to p<0.001, uncorrected for multiple comparisons, a few clusters surfaced. These comprised relative deficits in the thickness of the cortical mantle in the superior parietal, posterior cingulate, superior frontal and postcentral cortex, as well as some localized clusters of enlarged thickness in the frontal and temporal lobes (see Table 3).

**Table 3:** Cortical thickness comparisons adult sample.

Contrast	Cortical region	Hemisphere	Talairach			Size	p_max_
			X	Y	Z	(mm^2^)	
**All ADHD vs Control**							
Control>ADHD	Superior Parietal	R	15.7	−72.9	43.6	90.84	2.10E-04
	Superior Frontal	R	10.8	6.1	38.3	3.85	7.19E-04
	Posterior Cingulate	L	5.42	−1.2	−16.1	5.42	2.68E-04
	Postcentral	L	−35.3	−24.1	46.3	8.72	3.12E-04
Control<ADHD	Temporal Pole	L	−43.7	12.4	−31.0	102.30	4.18E-04
	Orbitofrontal	R	29.1	20.8	−21.3	39.22	4.96E-04
	Pars Opercularis	R	53.5	14.9	2.3	24.62	6.78E-04
**Medicated ADHD vs Control**							
Control>ADHD	Superior Parietal	R	15.6	−72.6	42.5	220.93	3.03E-05
	Inferior Parietal	L	−45.3	−69.4	18.6	14.66	5.81E-04
	Precentral	L	−33.3	−24.6	46.7	10.31	1.43E-04
Control<ADHD	Orbitofrontal	R	46.5	27.9	−9.3	167.56	2.04E-04
**Medicated ADHD vs Medication-Naïve ADHD**							
Med.Naïve>Med.ADHD	Inferior Parietal	L	−59.6	−26.2	36.8	16.36	7.08E-04
		R	41.1	−57.1	18.4	85.09	3.56E-05
	Superior Frontal	R	22.0	39.0	35.6	21.79	5.47E-04
	Mid-Cingulate	L	−14.5	−34.2	46.4	2.87	8.39E-04
	Lingual	L	−21.6	−81.1	−6.0	24.92	6.70E-04
	Fusiform	R	26.8	−68.5	−4.3	25.90	4.01E-04
	Lateral Occipital	R	28.6	−96.1	1.1	2.53	9.82E-04
Med.Naïve<Med.ADHD	**-**						

Full results of the ‘control>ADHD’ and ‘control<ADHD’ comparisons involving the complete adult ADHD sample (a combination of the medication-naïve (Med.Naïve ADHD) and medicated adult ADHD groups (Med.ADHD), as well as the comparisons including only the medicated patients with ADHD. The peak value of the posterior cingulate cortex cluster is actually located in the dorsal part of the corpus callosum directly adjacent to the posterior cingulate cortex, although the cluster extends primarily to the posterior cingulate cortex. The last section of the table represents the differences between the medication-naïve ADHD group and the medicated ADHD patients. Results are thresholded at p<0.001 uncorrected. No results were obtained for these comparisons at a threshold of p<0.05 MCTSB corrected for multiple comparisons.

**Figure 2:**
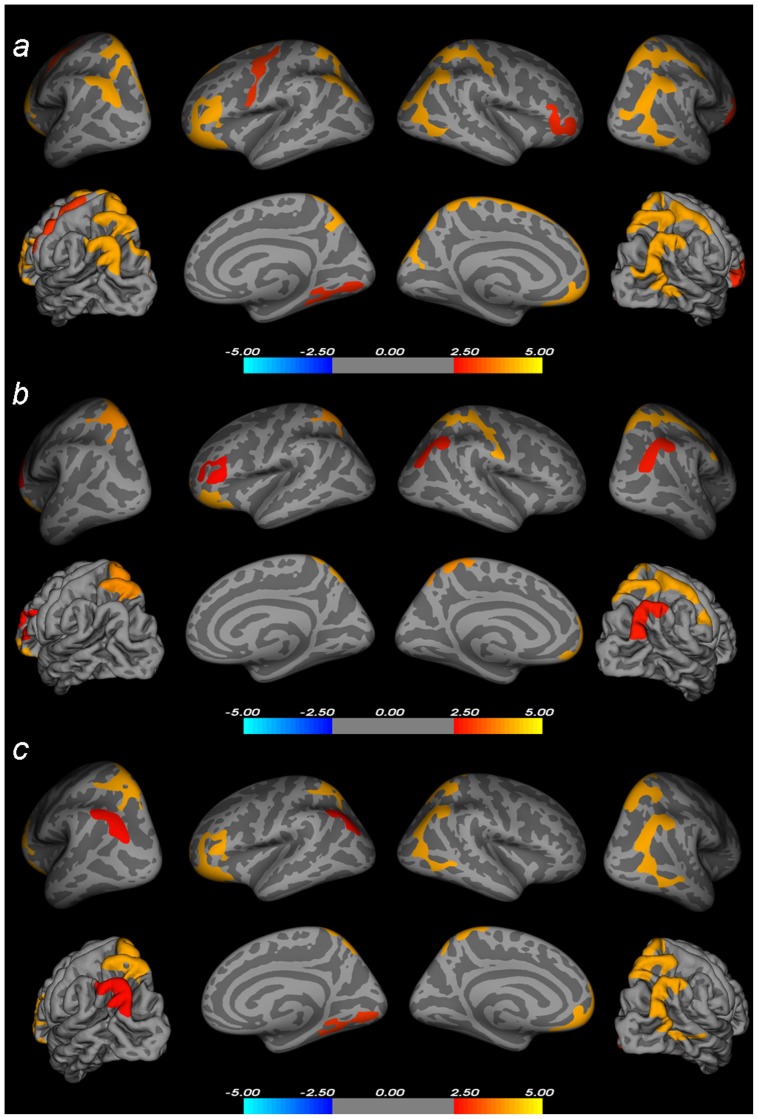
Statistical maps of the interaction contrasts. A) Diagnosis (ADHD/control)×Age Group (adult/child) interaction including the whole sample, overlaid on inflated and pial brain surfaces (colors encode the −log10(p-value)). B) Results of the interaction contrast on the samples excluding the medication-naïve participants from all groups, overlaid on inflated and pial brain surfaces (colors encode the −log10(p-value)). C) Results of the interaction contrast on the samples excluding all female participants from the sample, overlaid on inflated and pial brain surfaces (colors encode the −log10(p-value)). The results are thresholded at p<0.05, MCTSB corrected for multiple comparisons.

To investigate whether child and adult patients with ADHD show a distinct neuroanatomical profile, we included all subjects into one model and applied an interaction contrast (Diagnosis×Age group). This comparison rendered widespread clusters across the cortical mantle (L inferior parietal/superior parietal cortex: Talairach −35.5 −77.7 30.5, 5324 mm^2^, p = 3.35E-07. R inferior parietal/lateral occipital/inferior temporal cortex: Talairach 39.3 −75.9 29.2, 2877.71 mm^2^, p = 9.20E-10. R superior parietal/postcentral cortex: Talairach 10.5 −63.4 54.2, 3103.33 mm^2^, p = 2.81E-05. R lingual/fusiform cortex: Talairach 10-5 −81.5 −1,7, 1386.22 mm^2^, p = 3.43E-04. L orbitofrontal/rostral middle frontal/inferior frontal cortex: Talairach −32.7 25.0 −16.3, 5540.25 mm^2^, p = 2.40E-05. R orbitofrontal/rostral middle frontal/inferior frontal cortex: Talairach 38.7 50.3 −7.2, 1369.08 mm^2^, p = 1.69E-04. L precentral cortex: Talairach −38.9 −5.1 53.0, 1394.64 mm^2^, p = 2.61E-05), which are depicted in Figure 2A.

To assess whether these findings do not primarily reflect the differences between the child and adult sample in the distribution of medication-naïve patients, we repeated our comparisons using only the medicated patients, which represent the sample most commonly used for neuroimaging studies on ADHD. When performing the interaction contrast excluding the medication-naïve patients in each age group (24 of the adult patients and 5 of the child patients), we observed similar results to those obtained with the whole sample (L superior parietal cortex: Talairach −20.6 −54.3 60.7, 1896.79 mm^2^, p = 4.07E-05. R inferior parietal cortex: Talairach 37.8 −75.4 30.8, 1319.94 mm^2^, p = 1.71E-07. R superior parietal/postcentral cortex: Talairach 52.8 −15.7 44.8, 2859.63 mm^2^, p = 4.07E-05.L pars triangularis: Talairach −45.9 30.9 7.2, 1183.80 mm^2^, p = 1.08E-04. L orbitofrontal cortex: Talairach −33.8 22.4 −17.1, 2213.20 mm^2^, p = 3.88E-04). Statistical maps depicting these results are displayed in Figure 2B. When repeating the two sample t-test comparing the control and ADHD group in the child sample, we observed clusters of thinner cortical surface in the same sections of the frontal, parietal and occipital cortex (see Table 2). No results were obtained for the contrast ‘Control<ADHD’. In the adult sample, a comparison of the ADHD and control groups including just those subjects with a history of stimulant treatment rendered, in line with the previous results, no results when applying a correction for multiple comparisons (p<0.05 MCTSB corrected for multiple comparisons). Only when lowering the threshold to p<0.001 (uncorrected for multiple comparisons) we observed localized deficits in the thickness of the cortical surface in the inferior and superior parietal cortex and precentral gyrus, as well as a localized cluster of thicker cortex in the right pars orbitalis (see Table 3).

To further examine the effects of stimulant medication on the cortical surface, we also directly compared the adult patients with a history of stimulant treatment to those patients who had never received any pharmacological treatment for their condition. No results were obtained for this comparison when applying a correction for multiple comparisons. At a threshold of p<0.001 uncorrected, we observed several small clusters where medication-naïve patients exhibited a significantly thicker cortical mantle in comparison to subjects exposed to stimulant medication, including some of the regions observed in the previous comparisons (see Table 3). No clusters of relatively enlarged laminar thickness were observed in patients with a history of pharmacological treatment compared to never-medicated patients.

As the child patient and control groups included several girls, we wanted to examine the relation between ADHD and gender. Therefore, to search for effects of gender in the ADHD and control group besides the normal differences between male and female brains, we applied an interaction contrast on the child sample (‘Diagnosis×Gender’). At a threshold of p<0.05 MCTSB corrected for multiple comparisons, no results were observed. For completeness, we also checked the results of this interaction effect using a more liberal threshold of p<0.001 uncorrected. At an uncorrected threshold, we found several regions where neurotypically developed boys show larger cortical thickness values than neurotypically developed girls, while this pattern was not observed in boys and girls with ADHD (L middle temporal cortex: Talairach −65.5 −20.0 −9.2, 332.92 mm^2^, p_max_ = 8.70E-06. L superior temporal cortex: Talairach −58.5 4.5 −6.3, 366.01 mm^2^, p_max_ = 3.12E-05. Talairach −58.7 −18.4 4.4, 34.26 mm^2^, p_max_ = 2.30E-04. L middle frontal cortex: Talairach −42.0 11.1 44.1, 181.30 mm^2^, p_max_ = 8.90E-06. Talairach −34.3 33.9 27.7, 14.03 mm^2^, p_max_ = 7.57E-04. R medial orbitofrontal cortex: Talairach 7.2 32.4 −10.3, 11.71 mm^2^, p_max_ = 5.59E-04) as well as some clusters showing the opposite pattern (R superior frontal cortex: Talairach 25.0 9.8 44.6, 45.30 mm^2^, p_max_ = 2.98E-05. Talairach 4.0 39.2 38.6, 20.64 mm^2^, p_max_ = 6.89E-04. R medial orbitofrontal cortex: Talairach 3.4 47.6 −23.0, 135.87 mm^2^, p_max_ = 2.40E-05).

To ensure that the observed differences in cortical thickness between the child and adult groups were not driven primarily by the divergent gender distribution in these samples, we repeated the interaction contrast including only the male participants in all groups (excluding 21 subjects: 13 girls from the child control group and 8 girls from the child ADHD group) (L inferior parietal cortex: Talairach −34.9 −77.0 31.5, 1203.01 mm^2^, p_max_ = 3.79E-07. L inferior parietal/lateral occipital/inferior temporal cortex: Talairach39.3 75.9 29.2, 2285.74 mm^2^, p_max_ = 1.69E-07. L superior parietal cortex: Talairach −7.0 −79.2 34.3, 1086.13 mm^2^, p_max_ = 4.52E-04. R superior parietal cortex: Talairach 10.7 −63.7 54.1, 1868.54 mm^2^, p_max_ = 5.19E-05.L orbitofrontal cortex: Talairach −33 7 34.6 −8.7, 4763.11 mm^2^, p_max_ = 9.31E-06. R lingual/fusiform cortex: Talairach 9.5 −82.0 −1.6, 1450.22 mm^2^, p_max_ = 1.81E-04.). The results of these comparisons are depicted in Figure 2C.

## Discussion

In this study, we observed substantial differences in laminar cortical thickness between patients diagnosed with ADHD and control subjects. These differences were primarily located in the bilateral inferior and superior parietal cortex, where patients with ADHD had a substantially thinner cortical mantle in comparison to control subjects. Further deficits were observed in parts of the frontal cortex (primarily in the orbitofrontal cortex and a frontal section covering the bilateral superior frontal and precentral cortex) and the bilateral medial occipital cortex.

Some previous studies have investigated cortical thickness in patients with ADHD, observing widespread reductions in cortical thickness. These deficits were mainly located in sections of the parietal and frontal cortex associated with attentional processing [13], [16]–[18], [20], [26], [27], [39]. The clusters observed in our study when comparing the patient and control groups correspond to regions that surfaced in previous studies of cortical thickness evaluating ADHD samples. For instance, Shaw et al. observed reduced cortical thickness in children with ADHD in several frontal regions, including the precentral cortex, the medial frontal and superior frontal cortex [18]. Accordingly, regions in the prefrontal cortex showed the most pronounced delay in attaining the thickness levels associated with brain maturation [20]. The superior frontal cortex, one of the main regions of deficient cortical thickness in our study, has previously been identified as a region characterized by substantial laminar thickness deficits in children and adults with ADHD [16]. Likewise, cortical abnormalities in components of the sensorimotor cortex such as the precentral gyrus have also been observed in previous studies [13], [17], [28]. The parietal cortex has also repeatedly surfaced as one of the main loci of cortical thickness deficits in patients with ADHD [13], [16]–[18], [26], [27]. In fact, the right parietal cortex was the only section of the brain showing a normalization of laminar thickness measures in children with a better clinical outcome in a neuroimaging study following the progress of ADHD children across development [18].

Only a few previous studies have applied an analysis of cortical thickness to evaluate cortical morphometry in adults with ADHD, and these, although primarily using mixed samples of medicated and medication-naïve patients, also point to deficits in regions that subserve attention and executive functions [13], [16], [26], [27]. Makris et al. found clusters of reduced cortical thickness in several brain areas dedicated to attentional processing, including regions in the right lateral inferior parietal cortex and temporo-occipital junction such as the angular and supramarginal gyri [26]. Likewise, in a prospective follow-up study examining adult patients diagnosed in childhood, a thinner cortical mantle was predominantly observed in regions associated with attentional processing within the frontal cortex (including the superior frontal and precentral gyri), the temporal and parietal cortex [13]. Almeida et al. examined the frontal lobe in a cross-sectional sample of children, adolescents and adults with ADHD and observed that the patient group, independent of age, showed a thinner cortical mantle in the superior frontal cortex in comparison to controls [16]. In a whole-brain analysis of this sample they primarily observed regions of thinner cortex in the fronto-parietal cortex [27]. In contrast, clusters of increased thickness surfaced in sensori-motor regions in the analyses of Duerden et al. [28].

When inspecting our results, a remarkable similarity to the fronto-parietal attention networks, especially the dorsal attention network, is immediately evident. Corbetta et al. proposed that attention is subserved by two partially functionally and anatomically segregated neural circuits that reflect a distinction between goal-directed top-down and stimulus-driven orienting [40]–[43]. Subsequent neuroimaging and electrophysiological studies have corroborated this account [43]–[45]. The dorsal fronto-parietal network is recruited by top-down attentional control, which directs attentional resources towards specific aspects of stimulus processing [40]–[43], [46]–[48]. This network centers on the frontal lobe near the precentral and the posterior tip of the superior frontal cortex, while parietal activity is distributed in and around the intraparietal sulcus and superior parietal lobe. The dorsal fronto-parietal network interacts with extrastriate regions of the ventral visual system such as the lingual gyrus and lateral occipital cortex to enhance visual processing and target detection via fronto-parieto-occipital projections.

The ventral attention network is more ventrally positioned within the brain and more strongly lateralized to the right hemisphere. This network is centered on the temporo-parietal junction (comprising inferior parts of the inferior parietal lobule and posterior parts of the temporal lobe, such as the supramarginal and superior temporal gyri) and the ventral frontal cortex (including the inferior and middle frontal cortex and the frontal operculum). In contrast to the dorsal system, this network supports stimulus-driven attention, and is specialized for the detection of behaviorally relevant stimuli, especially unattended or low-frequency events [40]–[43].

The regions that harbor the cortical differences between ADHD patients and control subjects observed in our study correspond to structures implicated in these attentional networks, especially the dorsal fronto-parietal attention network. In fact, the pattern of regions showing the most severe deficits in cortical thickness compared to control subjects bears a remarkable resemblance to the attention networks as described by previous studies (see for instance [40]–[43]). The detection of substantial cortical deficits in brain regions subserving attentional functions is in agreement with the core role that symptoms of inattention play in the disorder [49].

In our study, we included both child and adult subjects. The inclusion of both an adult and a child sample in the same statistical model is important to shed light on the debate on whether ADHD represents a delay in cortical maturation or a persistent deviation from normality. In the present study, the children with ADHD showed very robust declines in cortical laminar thickness in comparison to control children. In fact, the anomalies in the child brain are so substantial that they survive a very restrictive p<0.001 MCTSB correction for multiple comparisons. In the adult sample, however, we observed no differences in cortical thickness between the patient and control groups. Only when applying a threshold uncorrected for multiple comparisons subtle deviations in laminar thickness between adult ADHD and adult control subjects surfaced. Although the same regions seem to be affected by the disorder in the child and adult forms of the disorder, in adult patients the deviations in laminar thickness are much less evident and only detectable at an uncorrected threshold. When performing an interaction contrast between diagnosis and age group, we observed highly significant clusters in the bilateral inferior and superior parietal cortex, the orbitofrontal cortex, the paracentral lobule and the lingual cortex. These results support the notion of a more pronounced laminar thickness deficit in children with the disorder, while the cortical mantle of adult patients is characterized by a more typical laminar thickness.

These findings seem to be in accordance with a delay in brain maturation that normalizes with age, as proposed by the maturation delay hypothesis [18], [20]. Indeed, previous studies have reported that certain anatomical deficits associated with ADHD can disappear during adolescence [18], [20], [50] Likewise, ADHD patients only partially develop the typical neuroanatomical asymmetry in cortical thickness associated with a maturing brain [22], and ADHD patients show a delay in attaining the peak cortical thickness throughout the cerebrum [20]. Interestingly, in the latter study, the right parietal cortex was the only region showing a normalization with age in children with a better outcome [20]. In typically developing children, this posterior component of the attentional network is thought to continue developing throughout adolescence and only fully mature in adulthood [51]. It should be noted, however, that these longitudinal studies did not control for the confounding effects of stimulant medication on the brain and did not examine the progress of these morphological measures into adulthood. Nonetheless, our main clusters of deficient laminar thickness when comparing ADHD patients with control subjects were located in these slowly developing cortical areas that were previously found to show a normalization with age in patients with a relatively better clinical outcome.

In our study, however, although the deficits in cortical thickness were relatively small in adult patients with the disorder, ADHD symptomatology was still present, suggesting that a relative normalization of neuroanatomical characteristics is not necessarily accompanied by symptom relief. It has been suggested that symptom remission may result from the compensatory maturation of other brain regions, such as the prefrontal cortex and cerebellum [13]. As adult patients with the disorder in our study also demonstrated deficits in these same parietal regions, although not to the same extent, we can speculate that the involved parietal brain regions have not reached full maturity in these patients whose symptoms continued into adulthood or did not receive sufficient compensatory support from other brain regions to attain symptom remission. Altogether, our observations suggest that patients suffering from ADHD in childhood are characterized by a widespread and substantial deficit in the cortical mantle. Persistent adult ADHD patients show a relatively typical laminar thickness across the cortical mantle, however, in spite of remaining symptomatic in adulthood.

Especially when investigating adult patients, it is challenging to recruit a sufficient number of patients who - although symptoms commenced in childhood- have never received pharmacological treatment for their condition. Therefore, most previous cortical thickness studies have not accounted for the effects of exposure to stimulant medication, and investigated mixed samples of medicated and unmedicated patients, although one research group has excluded stimulant treatment as a confounding factor by using a group of adult medication-naïve patients [16], [27]. After evaluating all incoming patients at the psychiatry section of Vall d'Hebron hospital over a 4-year period, we were able to acquire brain MRI scans of a relatively substantial group of medication-naïve adult patients with ADHD in addition to the more common group of patients with a history of stimulant drug exposure. When performing the comparisons across the diagnostic groups including just the patients with a history of stimulant treatment, we observed results that were very similar to those obtained using the conjoined sample. However, when separately comparing the sample of adults with ADHD who had never received any medication for their condition to the patients with a history of stimulant treatment, no statistically significant differences in cortical laminar thickness were observed. Only when substantially lowering the applied threshold we observed small clusters of thicker cortex in frontal, parietal and occipital regions. These results suggest that exposure to stimulant drugs is not associated with robust changes in laminar thickness across the cortical mantle. It should be noted, however, that these comparisons could only be performed in the adult sample, as the group of medication-naïve children included in our study was not sufficiently large for a meaningful analysis. Future cortical thickness studies using a more substantial group of medicaton-naïve children with ADHD might be able to isolate the effects of the disorder from the impact of previous exposure to stimulant drugs.

In sum, when comparing the laminar cortical mantle between patients with ADHD patients and control subjects, we observed widespread reductions in cortical thickness in the bilateral parietal cortex (especially the inferior and superior parietal gyri), the bilateral frontal cortex (primarily covering the superior frontal and precentral gyri as well as a section of the orbitofrontal cortex) and the bilateral medial occipital cortex (the lingual gyrus), showing a remarkable resemblance to the neural networks for attentional processing as described in the literature. The deficits in the cortical surface were very pronounced in the child patients. In the adult patients, however, a more typical laminar thickness was observed across the cortical mantle. These findings indicate that childhood ADHD is associated with robust deficits in the cortical mantle, which are especially prominent in brain structures subserving attentional processing.
