# Correction: Laminar Thickness Alterations in the Fronto-Parietal Cortical Mantle of Patients with Attention-Deficit/Hyperactivity Disorder

**DOI:** 10.1371/annotation/7f26a4d8-ec45-4d43-a388-f2d0d4f1a76a

**Published:** 2013-07-09

**Authors:** Elseline Hoekzema, Susana Carmona, J. Antoni Ramos-Quiroga, Vanesa Richarte Fernández, Marisol Picado, Rosa Bosch, Juan Carlos Soliva, Mariana Rovira, Yolanda Vives, Antonio Bulbena, Adolf Tobeña, Miguel Casas, Oscar Vilarroya

There is an error in the presentation of author Vanesa Richarte Fernández' name in the citation. The correct abbreviation is: "Richarte Fernández V". 

